# Conservative Surgery

**Published:** 1867-12

**Authors:** C. F. Hart


### CONSERVATIVE SURGERY.

By C. F. HART, M.D.

In some medical journal, a few days since, I found the question, Is not the knife too seldom used? which might very appropriately call forth an answer by another question, Is not the knife too frequently used? I think it is.

You, no doubt, as well as others, have seen the many accounts in the papers of late, headed false diagnosis in surgery. During the late war, many a poor soldier lost his limb from this cause, whilst others, by the desire of the surgeon to gain some reputation with the knife.

In the summer of 1860, July I think, Dr. E. R. Cook, of Kentucky, called at my office, and laid the following case before me, and asked my opinion as to the propriety of amputating:—The patient, a boy about 14 years of age, had, the day before, whilst out hunting, accidentally shot himself, the contents of the gun having entered the right arm, as the Dr. said, at the wrist joint, breaking up the articulations, and carrying away from 1½ to 2 inches of radius and ulna, the head of the bones shattered. From the statement, my opinion was, that amputation was inevitable, and the sooner the better, but that I could not give a satisfactory answer without seeing the case; that I thought the authors on the subject would sustain him in the operation; that where the heads of the bones were shattered, and the articulations broken up, it was a proper case for amputation.

He then proposed that I would meet him and others in the case. I consented, and upon examination of the patient, found that the charge had entered the arm about ½ an inch to 1 inch from the head of the bones, and between them, missing the radial artery, but carrying away, as he had stated, about 1½ or 2 inches of the bones. I directed him to work his fingers, which he did, and could move the thumb, index, and little fingers with ease, the ring and index finger could scarcely be moved, and caused a good deal of pain; the head of the bones, so far as we could judge, was sound. I, of course, changed my former opinion, and advised cold water dressing, or irrigation of ice water, and try and save the arm. Some were in favor of immediate amputation. My reasons, as then expressed, for opposing such a course, was, that the patient was young, healthy, and robust; the periphery was intact; the heads of the bones, so far as we could discover, were uninjured. Dr. Cook thought as I did, and finally the others; the result was, the recovery of the patient, with a good and useful arm.

Case II. During the war, one of the soldiers, in the hurry and confusion of a retreat, let his pistol fall, which caused it to fire, the ball taking effect in the left arm, about 2½ inches from the joint, passing through and carrying away from 1 to 1½ inches of both bones; this was in August, 1864; he was left in charge of Dr. Wm. Feland, then a citizen, but formerly Assistant-Surgeon of the 32d Illinois Infantry, I think; he asked me to see the case with him; the question then was, Would it not be necessary to amputate in order to save the patient's life? The weather was very hot, and gangrene strongly threatened. Some of the older physicians were for taking it off. The Dr. thought it might possibly be saved by close and careful attention. I agreed with him. The soldier was anxious that it should be saved if possible, and willing to suffer the pain as well as the danger of gangrene. He was young—not quite 20—healthy, and robust. The result was, the arm was saved, and he reënlisted and served to the end of the war.

Case III. In October of the same year, during an engagement, one of the boys was wounded through the left arm, about

1½ inches from the joint, with a large minnie ball, passing through and carrying with it not less than 2 inches of both bones. I did nothing with it that day, but throw a loose bandage wet with cold water over it, and gave him some morphine, with directions to keep it wet until I could give him further attention. The next day, I had several of the physicians of the place with me, and when we came to this young man, they were all in favor of amputating, which I could not consent to. The boy was young, in fine health, and good game, and I thought, with proper care and attention, his arm might be saved, which proved to be true. He recovered, with the arm ½ an inch short, a little twisted, but with perfect use of it.

