# Correction

**Published:** 1868-03-15

**Authors:** 


*Correction.*—In the report of the proceedings of the society in the *Journal* of last December, the word "other" should be substituted for the word "these" in the following sentence: Drs. Davis and Fisher reported cases of delirium tremens under the care of *these* physicians, which had terminated suddenly (in death) after large and repeated doses of *Tinct. Digitalis* had been taken.

